# Fire Severity Controlled Susceptibility to a 1940s Spruce Beetle Outbreak in Colorado, USA

**DOI:** 10.1371/journal.pone.0158138

**Published:** 2016-07-20

**Authors:** Dominik Kulakowski, Thomas T. Veblen, Peter Bebi

**Affiliations:** 1 Graduate School of Geography, Clark University, Worcester, MA, United States of America; 2 WSL Institute for Snow and Avalanche Research SLF, Davos, Switzerland; 3 Department of Geography, University of Colorado, Boulder, CO, United States of America; Ecole Pratique des Hautes Etudes, FRANCE

## Abstract

The frequency, magnitude, and size of forest disturbances are increasing globally. Much recent research has focused on how the occurrence of one disturbance may affect susceptibility to subsequent disturbances. While much has been learned about such linked disturbances, the strength of the interactions is likely to be contingent on the severity of disturbances as well as climatic conditions, both of which can affect disturbance intensity and tree resistance to disturbances. Subalpine forests in western Colorado were affected by extensive and severe wildfires in the late 19^th^ century and an extensive and severe outbreak of spruce beetle (*Dendroctonus rufipennis*) in the 1940s. Previous research found that most, but not all, of the stands that burned and established following the late 19^th^ century fires were not susceptible to the 1940s outbreak as beetles preferentially attack larger trees and stands in advanced stages of development. However, previous research also left open the possibility that some stands that burned and established following the 19^th^ century fires may have been attacked during the 1940s outbreak. Understanding how strongly stand structure, as shaped by disturbances of varying severity, affected susceptibility to past outbreaks is important to provide a baseline for assessing the degree to which recent climate change may be relaxing the preferences of beetles for larger trees and for stands in latter stages of structural development and thereby changing the nature of linked disturbances. Here, dendroecological methods were used to study disturbance history and tree age of stands in the White River National Forest in Western Colorado that were identified in historical documents or remotely-sensed images as having burned in the 19^th^ century and having been attacked by spruce beetle in the 1940s. Dendroecological reconstructions indicate that in young post-fire stands only old remnant trees that survived the otherwise stand-replacing fires were killed in the 1940s outbreak. No young post-fire trees (< ca. 128 years) were susceptible to the 1940s outbreak, implying that under the relatively cool and wet conditions of the mid-20^th^ century, susceptibility to and spatial patterns of spruce beetle outbreak were most likely controlled by variations in severity of prior disturbance by fire. This study provides a baseline for comparing linked disturbances under the relatively warmer and drier conditions of recent (e.g. post-1990) outbreaks in order to assess how climate mitigates the degree to which pre-disturbance history and structure affect susceptibility to disturbances.

## Introduction

Disturbances such as wildfires and insect outbreaks are key drivers of the spatiotemporal patterns and processes of forest ecosystems. Even large and severe disturbances often leave surviving trees, which then can play an important role in promoting spatial heterogeneity in post-disturbance regeneration [1] and which can also affect other dynamics including susceptibility to subsequent disturbances. Much recent research has focused on how the occurrence of one disturbance can affect the occurrence of subsequent disturbances [e.g., 2–4]. Most studies of such linked disturbances have focused only on the presence or absence of disturbances, based on inconsistent thresholds of severity. There are recent exceptions that have tested how severity of beetle infestation affects subsequent fire severity [5–7]. However, scant research attention has been given to the question of how variability in severity of initial fires influences severity of subsequent bark beetle outbreaks. Furthermore, because the importance of pre-disturbance vegetation conditions on susceptibility to disturbances is theoretically expected to decrease as the intensity of subsequent disturbance increases, the strength of interactions among disturbances is likely to change as climatically-driven disturbances become more intense under climate change. To assess such potential climatically-driven changes in disturbance interactions it is important to establish a clear baseline of how disturbances have interacted under less extreme climatic conditions. Thus, to provide baseline comparisons with disturbance interactions occurring under the warmer, drier climate of the early 21^st^ century in western Colorado [8,9], here we assess how variability in high-severity fires affected susceptibility to an outbreak of spruce beetle (*Dendroctonus rufipennis*) in the 1940s in western Colorado.

As climate change is driving an increase in the intensity and likelihood of extreme weather events [10] as well as in the frequency, intensity, and size of forest disturbances such as fire and bark beetle outbreaks in the 21^st^ century [11,12], it is increasingly likely that any given ecosystem will be affected by more than one disturbance. Therefore it is critical to understand how the occurrence or severity of one disturbance affects the occurrence or severity of a second disturbance (i.e. linked disturbances; *sensu* [13]. For example, in Rocky Mountain subalpine forests, severe blowdown has been shown to amplify the severity of fire occurring a few years later [14] which then affects post-fire regeneration [15]. In contrast, stand-replacing fires have been shown to reduce the probability of blowdown for the subsequent c. 100 years [16], and spruce beetle outbreak has been shown to reduce the probability of a second outbreak for the subsequent c. 60 years [17]. Although there has been much interest in assessing how previous bark beetle outbreaks may influence subsequent fire severity [18–23], studies of the effects of previous fire severity on subsequent likelihood or severity of bark beetle outbreaks are scarce (but see [2,3].

Wildfires [24,25], outbreaks of bark beetles (*Dendroctonus spp*.; [26–28]), and the interactions among these disturbances [23] have long shaped the forests of the Rocky Mountains. The highest elevation subalpine forests in this region are dominated by Engelmann spruce and subalpine fir (*Picea engelmannii* and *Abies lasiocarpa*) and are characterized by large, high-severity fires, outbreaks of spruce beetle (*D*. *rufipennis*), as well as the interactions between these disturbances. Spruce beetle outbreaks are influenced by climate as well as stand structure and composition [29]. Wildfires, especially high-severity ones, can shape stand structure and tree size, which in turn affect susceptibility to bark beetles and other insects. Spruce beetles preferentially attack larger trees and stands in later stages of development [30]. As a result, for c. 100 years following stand-replacing fires, Engelmann spruce stands in Colorado have been shown to be less susceptible to attack by spruce beetle in the nineteenth [31] and twentieth [2,32,33] centuries, even when large and severe outbreaks killed most large spruce in surrounding stands. However, the constraints of tree and stand attributes on eruptions of bark beetle populations may be reduced under a warmer and drier climate as such conditions promote more rapid growth of beetle populations [34,12,29] and simultaneously decrease tree resistance to beetle attack [35,27,36]. For example, during a spruce beetle outbreak in the 21^st^ century numerous small-diameter and suppressed trees were attacked by spruce beetle prior to any eventual host saturation, suggesting that tree-level constraints had been relaxed [37]. However, under a future scenario of sufficiently dry climate, reduction of host tree populations may reduce probability of bark beetle outbreaks in some habitats [38]. To assess how climate change may affect how disturbances interact, it is essential to establish a clear baseline of these interactions under less extreme climatic conditions than those of the late 20^th^ and early 21^st^ centuries.

Wildfires in the subalpine forests of the Rocky Mountains have historically been large and severe events in which most canopy trees over extensive areas were killed [24,25]. Consequently, most research on how fires affect susceptibility to spruce beetle has focused on the effects of such severe, stand-replacing fires. However, even high-severity fires leave survivors that are important in post-disturbance dynamics [1]. How spatial variability in fire severity may affect the likelihood or severity of subsequent spruce beetle infestation has not previously been investigated. In this context, and given the critical effects of fires on subsequent disturbance regimes, it is important to examine the question of how variability in fire severity affects how fires interact with other disturbances.

Previous research conducted at stand, landscape and regional scales showed that high-severity fires in the late-19^th^ century had a dampening influence on subsequent spruce beetle infestation for periods of at least 80 years [2,32,33]. A broad-scale analysis based on historical documents and remotely-sensed data showed that stands that established after widespread fires in the late 19^th^ century were less susceptible to a widespread spruce beetle outbreak in the Flat Tops area of western Colorado in the 1940s [32]. Specifically, there was only a 5.5% overlap between the surface area burned in c. 1879 with the 1940s spruce beetle outbreak [32]. Overlapping areas of both disturbances were mostly at lower elevations, on northerly aspects, and on less steep slopes. Given the landscape-scale focus and coarse resolution of the Bebi et al. [32] study as well as the lack of fine-scale field data, it was not possible to determine the conditions that explained this partial overlap of the two disturbance types. This overlap may have been due to three non-mutually exclusive explanations: (1) rapid tree growth and stand development on favorable sites; (2) beetle pressure overcoming constraints related to stand structure attributes; or (3) spatial variation in severity of fires resulting in legacies of older trees either as scattered individuals or as remnant patches. The actual reasons for this variability are important because of their implication for our understanding of potential changes in disturbance interactions under climate change. Here we use tree-ring methods to examine tree ages, tree mortality and growth patterns in stands that on the basis of maps of fire history [39] and air photo interpretation [32] had burned in the late 19^th^ century but were still affected by the 1940s spruce beetle outbreak. We compare these stands to stands that burned in the 19^th^ century but were not affected by the outbreak and also to stands that did not burn in the 19^th^ century. Our key objective was to determine why some stands apparently affected by high-severity 19^th^ century fires were also affected by the 1940s spruce beetle outbreak, in contrast to the predominant pattern of non-overlap of these disturbances in c. 95% of the study area.

## Materials and Methods

### Study area

**Fig 1:**
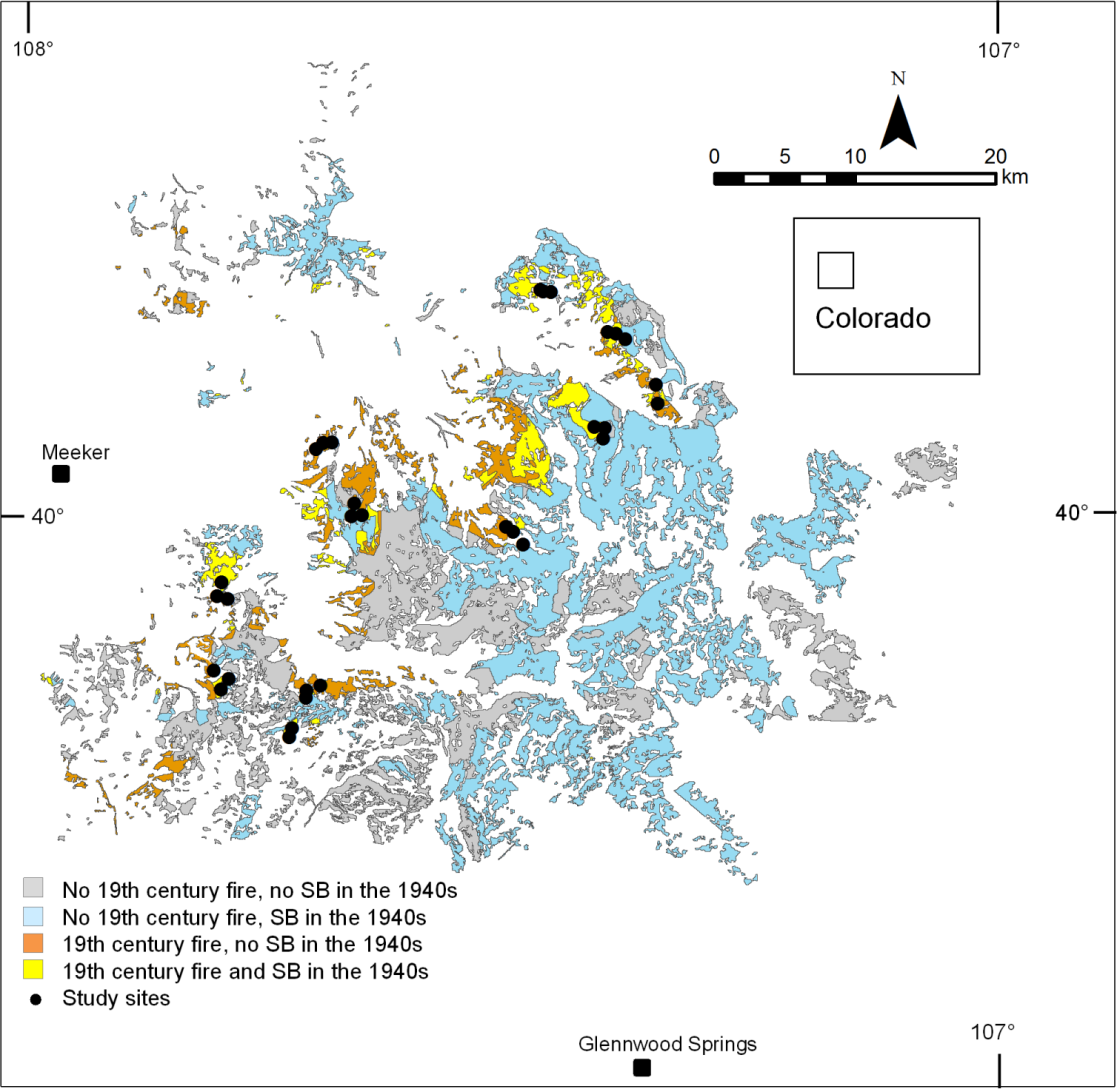
Location of study sites. Areas affected by the 19^th^ century fires and by the 1940s spruce beetle (SB) outbreak as reconstructed by Bebi et al. [32].

The study area is located in White River National Forest of northwestern Colorado (40°N, 107°W; Fig 1). Forests are dominated by *Picea engelmannii* (Parry) Engelm. (Engelmann spruce) and *Abies lasiocarpa* (Hook.) Nutt (subalpine fir). Elevation of study sites ranges from 2600 to 3100 m a.s.l. The closest weather stations with fairly complete data extending back to the 1930s are in Dillon, CO (2761 m asl; c. 100 km from the study area) and Hayden, CO (1963 m asl; c. 50 km from the study area). The records of these two climate stations were used for comparing climatic conditions during the 1940s spruce beetle outbreak with those during the outbreak of the early 21^st^ century. Climate parameters were selected for analyses that in previous studies have been shown to be predictive of spruce beetle outbreaks (i.e. minimum March and October temperatures, annual mean temperature, and annual precipitation; [40, 29]).

This area was affected by extensive and severe wildfires in the nineteenth century and an extensive and severe outbreak of spruce beetle (*Dendroctonus rufipennis* Kirby) in the 1940s [2,32,33]. Tree-ring dating of fire scars and post-fire cohorts document widespread burning at high severity of much of the study area in one or several fires centered on approximately 1879–1880 [2,33]. The area burned during these major fire years and through the 1890s was mapped in 1898 [39]. The study area was not affected by large recent (i.e., post 1908) fires [30, USDA Forest Service, Glenwood Springs, Colorado, USA, *unpublished data*).

A large spruce beetle outbreak affected most forests dominated by Engelmann spruce in northwestern Colorado in the 1940s, including most of the current study area. The outbreak was centered on White River National Forest where it killed > 90% of the spruce [41,42]. In the Bebi et al. [32] study, the extent of the 1940s spruce beetle outbreak was mapped from aerial photography (IR-1984, 1:50 000 and color-1971, 1:10 000) based on percentage of standing dead trees in the overstory, frequency of logs on the ground, and abundance of subcanopy trees (i.e. < 20 cm diameter at breast height). Young (c. 50–120 yr old) post-fire stands were identified by their characteristic dense and uniform canopies with small crown diameters [32]. This aerial photo interpretation was calibrated and verified by field observations and field data collection [32,33]. Stands significantly affected by spruce beetle attack were defined as stands in which > 30% of the canopy trees (standing plus downed) were dead. There were no other large spruce beetle outbreaks in Colorado until an outbreak that initiated in 1997.

### Field sampling

Field sampling was permitted by the USDA White River National Forest. Field sampling did not involve endangered or protected species. Based on a stratified random design of the disturbance maps produced by Bebi et al. [32], for the current study we selected 32 sites to include a range of stand structures and disturbance histories. Sites were stratified to include an equal number in each of three combinations of 19^th^ century fires and the 1940s outbreak: burned and unaffected by outbreak, burned and affected by outbreak, and unburned and affected by outbreak. Tree-ring samples were collected in June and July of 2001 following procedures previously used to reconstruct fire and spruce beetle history in similar subalpine forests in the same study area in western Colorado [43,2,33]. We collected 12 to 23 tree core samples from host and non-host species per site at all 32 sites, with a focus on detecting the 19^th^ century fires and the 1940s spruce beetle outbreak. A total of 522 tree core samples were collected. In subalpine forest stands in Colorado that have initiated after stand-replacing fires, stand age is a good approximation of the time since the last stand-replacing fire [44,2,16,33]. A sampling point was randomly located in each of the 32 sites. In order to obtain stand-origin dates, increment cores were collected from the largest live and dead trees within a c. 300 m search area around the sampling point. The number of trees cored depended on the difficulty of preliminarily assessing the disturbance history of each patch based on examination of tree cores in the field. If size structure of a stand was complex due to >1 unique cohort (e.g. a bimodal size structure), then cores were collected from trees of up to one additional cohort. Testing in similar forests has shown that subjectively selecting the largest trees gives a better estimate of time since fire than randomly selecting trees [44]. To reconstruct the spruce beetle outbreak additional cores were collected from dead spruce and nearby (i.e. within c. 5 m) non-host trees that may have shown a corresponding growth release. Selected trees were cored as close to the ground and to the pith as was possible. Tree age is reported as age at coring height which ranged from 11 to 120 cm (median = 38 cm). In order to assess the severity of the 1940s outbreak from percentage of standing dead spruce trees, in each patch five 10 x 10 m plots were located at 30-m intervals along a randomly located transect. Within each plot d.b.h. of all canopy trees (≥20 cm d.b.h.) was measured and species and status as living or dead was recorded for each tree. Because most Engelmann spruce killed by spruce beetle remain standing for many decades [41], the current density and basal area of dead-standing trees are good indicators of the amount of beetle-caused mortality. For example, based on monitoring of plots over c. 25 years, the spruce killed in the White River outbreak of the 1940s had been falling at the rate of 1.5% per year [42].

### Analytical methods

Standard procedures were used to process increment core samples [45]. In the event that the increment core sample missed the pith, a simple geometric model was used to estimate the number of rings to the pith [46]. This method was used to estimate a maximum of twenty missing years to the pith. Cores that missed the pith by > 20 years were counted as minimum ages. Tree age is reported as age at coring height. To date the establishment and mortality of dead trees and to address the problem of missing and false rings, cores were cross-dated visually using marker years [45] or were measured and cross-dated quantitatively using the program CDendro [47].

Disturbance reconstruction was focused on detection of severe disturbances by fire and beetle. Therefore, our interpretations of disturbance history were focused on synchronous establishment, mortality, and releases across a stand. Stand disturbance history was reconstructed based on stand-origin dates, dates of mortality of dead trees, and releases in live and dead remnant trees that survived the 1940s outbreak. No evidence of other extensive disturbances (e.g. uprooted trees oriented in the same direction that would indicate wind blowdown) was found in the study area.

Methods of reconstructing fire history follow methods previously used in these forests [e.g., 16,33]. Because fires that shape Colorado subalpine forests are primarily large, severe, and infrequent, our primary aim was to group stands into broad age classes that most likely arose following such fires. To do so, dates of establishment of the oldest trees in each stand were used as an approximation of the year of the last stand-replacing fire. The estimated fire date based on stand origin can be within as little as one year of the actual date for more recent fires (c. 130 years ago) and is typically more approximate (within a few years to several decades) for older fires (> 200 years ago) [44,48]. Old-growth stands typically have a broad range of ages among the largest trees in comparison with post-fire stands. The potentially long period of gap-phase dynamics in old-growth stands eventually exerts a more dominant effect on stand structure than the initial stand-replacing fire. In contrast, younger post-fire stands typically have identifiable pulses of establishment, even following mixed severity fires that can result in a mixed age structure within a stand. A pulse of establishment was defined as the earliest episode during which at least 30% of trees established within a 40 year period during the 18th century, at least 40% of trees established within a 40 year period during the 19th century, or at least 50% of trees established within a 40 year period during the 20th century. Thus, the criterion for detection of post-fire cohorts was scaled to reflect the disappearance of evidence over time.

Methods of reconstructing history of spruce beetle outbreak follow methods previously used in these forests [e.g., 43,33]. Outbreaks of spruce beetle typically result in coincident mortality of large (>10 cm d.b.h.) Engelmann spruce during the outbreak and coincident releases (abrupt >200% increases in ring width sustained >10 years) of the surviving trees [43]. The occurrence of the 1940s beetle outbreak was determined in each sampled stand by checking tree cores for coincident releases in all trees and dating the year of mortality of dead trees. The severity of the outbreak in each stand was estimated as the percent of ≥20 cm Engelmann spruce that were dead within the 100 m^2^ plots. Although this likely overestimates the severity of the outbreak, our data indicate that 77.8% of datable dead spruce in our plots died during the time of the outbreak (between 1938 and 1946), consistent with previous research that showed that most death dates of standing spruce in the region correspond with spruce beetle outbreaks [49]. Stands were categorized as having been affected by the 1940s outbreak if at least 33.3% of trees showed releases 1940–1959. Logistic regression was used to test probability of trees being killed during the outbreak as a function of tree age at the beginning of the outbreak (1939). Tree age is based on pith, estimated pith, and minimum ages of 306 Engelmann spruce across all 32 sites.

## Results

### Climate Comparison

**Fig 2:**
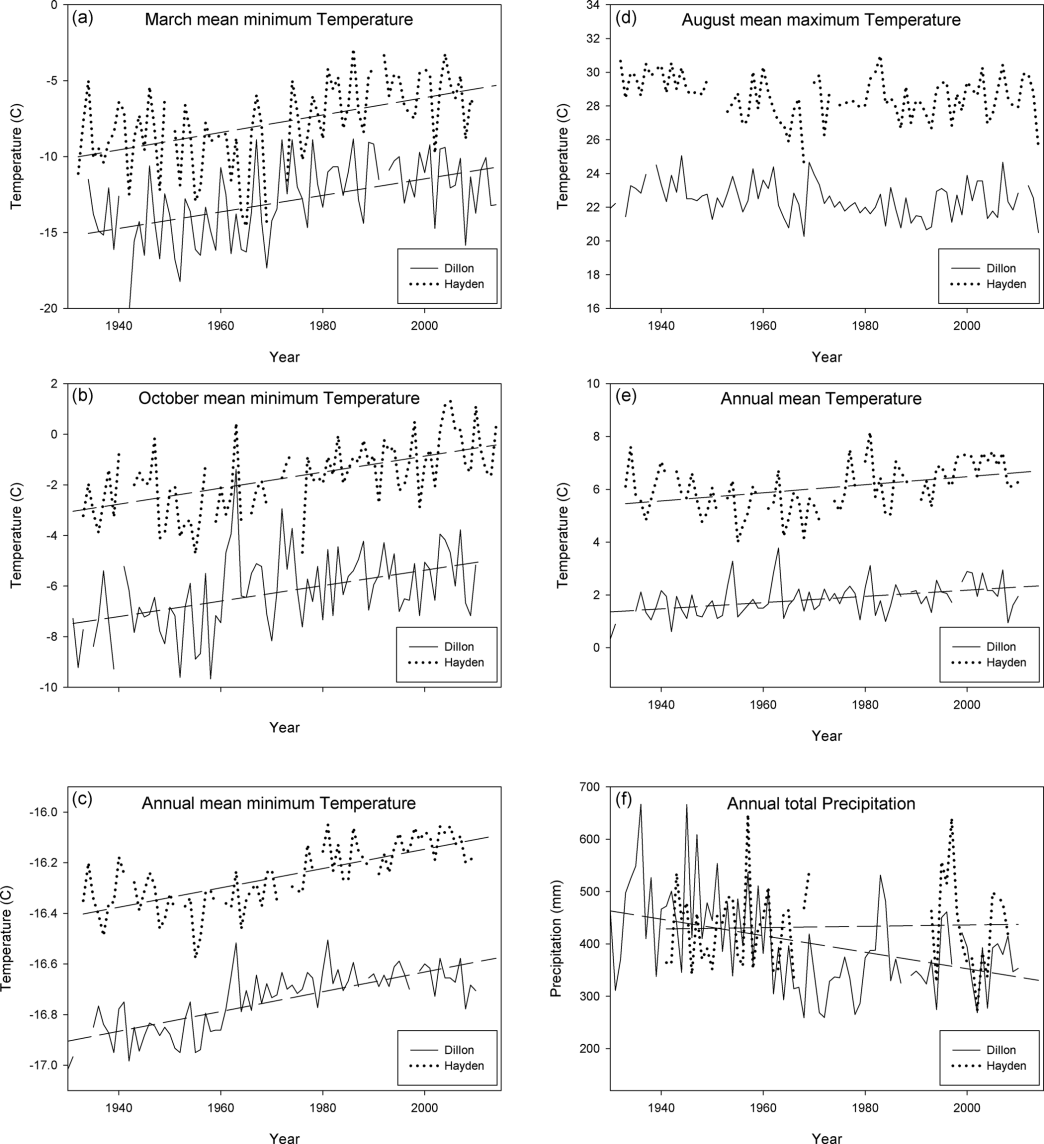
Climate trends. Temperature (°C) and precipitation (mm) during the 20^th^ century for Dillon, CO (2761 m asl) and Hayden, CO (1963 m asl). The wide dashed lines depict significant (*p* <0.001) trends between 1930 and 2014. Trends for August mean maximum temperature were not significant (*p* >0.01).

Both the Dillon and Hayden climate stations indicate substantial differences in climate conditions during the 1935–1949 spruce beetle outbreak and the early 21^st^ century (Table 1). Mean annual temperatures and minimum March and October temperatures are higher in the early 21^st^ century in comparison to the 1935–1949 period (Table 1). Annual, March and October minimum temperatures exhibit significant (*p* <0.001) upward trends between 1930 and 2014 (Fig 2). Annual mean temperatures also exhibit a smaller, but significant (*p* <0.001) upward trends between 1930 and 2014. Precipitation trends were different between the two stations, but the Dillon station that is at the same elevation as the study area indicates that the 1940s were also wetter than the early 21^st^ century (*two-tailed t-test p* < 0.01).

**Table 1:** Climate comparison. Summary mean temperature (°C) and precipitation (mm) data for the time periods of the 1940s and the 2000s outbreak for Dillon, CO (2761 m asl) and Hayden, CO (1963 m asl).

	1935–1949	2000–2014
Dillon Annual mean temperature***	1.5	2.3
Hayden Annual mean temperature ***	5.9	6.9
Dillon August mean max temperature ^ns^	23.2	22.6
Hayden August mean max temperature ^ns^	29.5	28.7
Dillon mean min March temperature **	-14.7	-11.7
Hayden mean min March temperature ***	-8.7	-5.8
Dillon mean min Oct temperature ***	-7.2	-5.3
Hayden mean min Oct temperature ***	-2.2	-0.4
Dillon mean min annual temperature ***	-16.9	-16.7
Hayden mean min annual temperature ***	-16.3	-16.1
Dillon mean annual precipitation**	486.0	367.1
Hayden mean annual precipitation **	384.5	472.9

*** *p* < 0.001

** *p* < 0.01

^ns^ *no significant difference based on two-tailed p-values of t-tests*

### Tree-ring reconstruction of disturbance history, tree mortality, and tree growth

**Fig 3:**
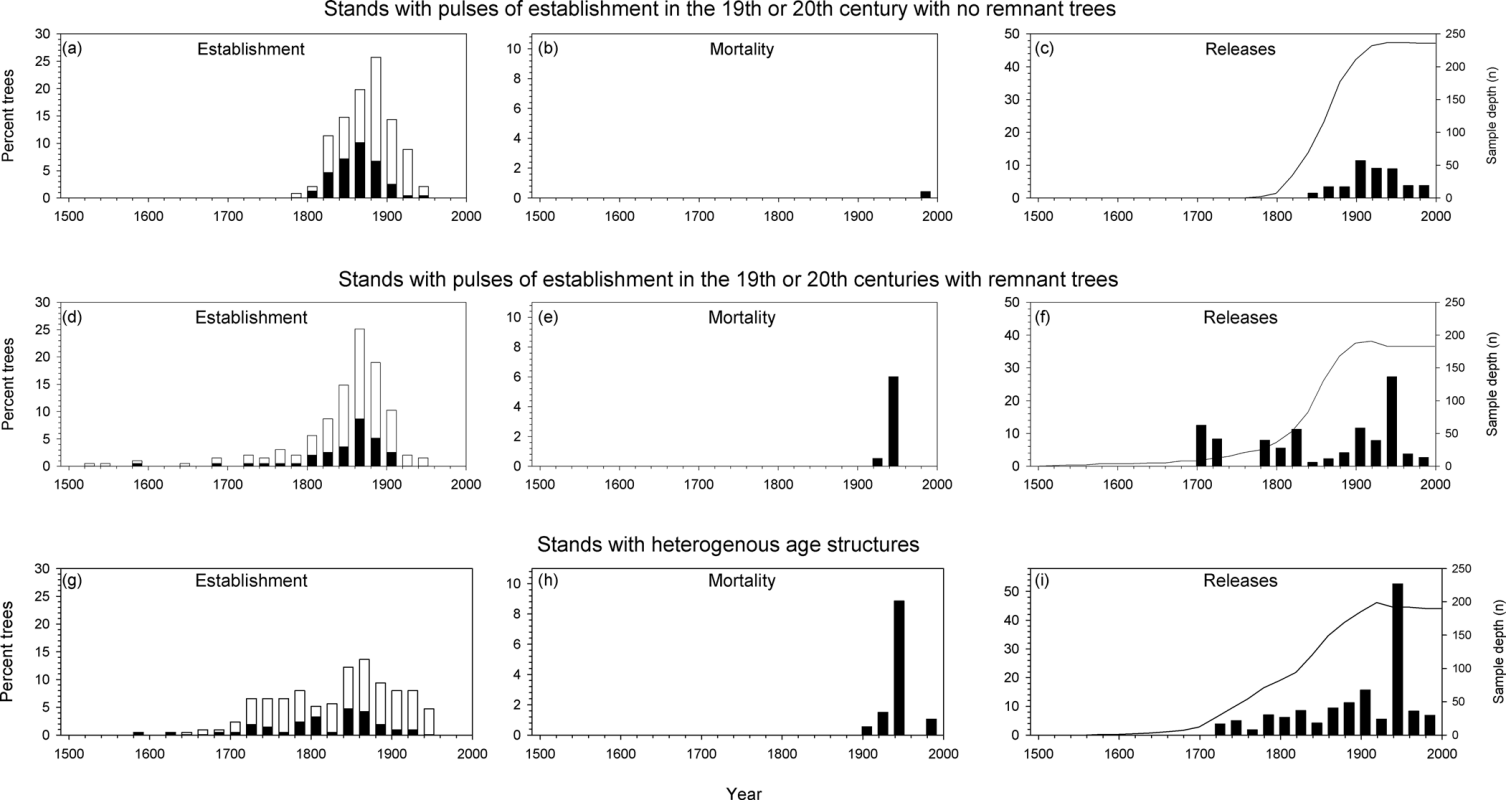
Stand disturbance histories. Dates of establishment expressed as a percentage of total trees established (black bars represent pith or estimated pith dates and gray bars represent minimum ages), mortality expressed as a percentage of total trees alive at the time (last complete year of growth), and releases expressed as a percentage of total trees alive at the time (abrupt > 200% increases in ring-width growth sustained > 10 years) of trees in 20 year bins in (a-c) stands characterized by a pulse of establishment in the 19^th^ or 20^th^ century with no remnant trees, data aggregated from 11 stands; (d-f) stands characterized by a pulse of establishment in the 19^th^ or 20^th^ centuries with remnant trees, data aggregated from 9 stands; and (g-i) stands characterized by heterogeneous age structures, data aggregated from 12 stands.

Of the 522 increment core samples corrected, 499 (95.6%) were successfully processed. Disturbance history was reconstructed based on successfully processed increment cores from these 499 trees (mean of 16; range of 9 to 20 cores per site) and recorded attributes (species, dbh, live vs. dead, etc.) of 4384 trees across 32 stands (Table 2; Fig 3). Stands were grouped into three general categories of fire history: stands characterized by (1) a single main pulse of establishment in the 19^th^ or 20^th^ century and no remnant older trees (i.e., no establishment dates earlier than c. 1800; n = 11); (2) a single main pulse of establishment in the 19^th^ century along with remnant older trees (i.e., including establishment dates earlier than c. 1800; n = 9); and (3) heterogeneous age structures with a high proportion of trees that established prior to the 19^th^ century (i.e., including establishment dates earlier than c. 1800; n = 12) (Fig 3).

**Table 2:** Stand age, structure, composition, and disturbance. Stand structure and composition (stems/ha) based on inventory of trees ≥ 10 cm dbh. A *pulse* of establishment was defined as the earliest episode during which at least 30% of trees established within a 40 year period during the 18^th^ century, at least 40% of trees established within a 40 year period during the 19^th^ century, or at least 50% of trees established within a 40 year period during the 20^th^ century. Stands were categorized as having been affected by the 1940s spruce beetle outbreak (SB) if at least 33.3% of trees showed releases in 1940–1959.

Site	Earliest establishment	Pulse (Century)	Remnant trees	Spruce/ha	Dead spruce /ha	Spruce (%)	Dead spruce (%)	Trees releasing 1940–1959 (%)	1940s SB
*Stands characterized by heterogeneous age structures*									
1B	1592	-	-	1540	620	74.0	40.3	73.3	Y
4B	1729	-	-	1260	1020	63.0	81.0	100.0	Y
2B	1800	-	-	1240	800	56.4	64.5	93.3	Y
3B	1677	Mid 18th	Y	1080	740	50.5	68.5	88.9	Y
11C	1722	-	-	1100	540	47.0	49.1	38.9	Y
4C	1631	-	-	1040	700	44.4	67.3	56.5	Y
11B	1796	-	-	760	520	40.9	68.4	42.1	Y
6B	1725	-	-	860	560	40.2	65.1	38.9	Y
1C	1735	Late 18^th^	Y	640	480	32.0	75.0	40.0	Y
11A	1708	-	-	520	440	24.8	84.6	57.9	Y
4A	1703	-	-	400	280	16.5	70.0	40.0	Y
8B	1750	-	-	100	100	6.2	100.0	21.0	N
*Stands characterized by a pulse of establishment in the 19*^*th*^ *or 20*^*th*^ *century with remnant trees*									
3C	1763	Mid 19^th^	Y	1820	1020	77.1	56.0	38.5	Y
5C	1656	Late 19^th^	Y	1200	480	67.4	40.0	45.8	Y
2C	1760	Mid 19^th^	Y	940	280	60.3	29.8	76.5	Y
6C	1794	Mid 19^th^	Y	1120	520	44.4	46.4	52.3	Y
10A	1684	Late 19^th^	Y	640	380	34.0	59.4	33.3	Y
8C	1808	Late 19^th^	Y	420	360	18.3	85.7	3.3	N
5A	1581	Early 19^th^	Y	160	120	9.4	75.0	12.5	N
7C	1755	Mid 19^th^	Y	180	160	6.7	88.9	6.3	N
5B	1533	Late 19^th^	Y	40	0	2.0	0.0	0.0	N
*Stands characterized by a pulse of establishment in the 19*^*th*^ *or 20*^*th*^ *century with no remnant trees*									
2A	1907	Early 20^th^	-	1620	860	56.6	53.1	0.0	N
1A	1790	Early 19^th^	-	533	100	37.6	18.8	25.0	N
9C	1802	Early 19^th^	-	360	240	18.4	66.7	13.3	N
10B	1858	Late 19^th^	-	140	20	11.7	14.3	4.2	N
8A	1874	Late 19^th^	-	160	0	11.4	0.0	4.3	N
7B	1841	Mid 19^th^	-	60	40	3.7	66.7	9.5	N
3A	1829	Late 19^th^	-	100	75	3.6	75.0	12.5	N
7A	1834	Mid 19^th^	-	40	40	2.2	100.0	4.8	N
9B	1807	Mid 19^th^	-	40	40	2.1	100.0	14.3	N
6A	1797	Late 19^th^	-	0	0	0.0	n/a	0.0	N
9A	1824	Mid 19^th^	-	0	0	0.0	n/a	4.0	N

Coincident mortality and releases, along with previously published data [2,32,33] indicated widespread outbreak across the study region in the 1940s, and this outbreak was strongly determined by the age structure of stands. At the stand scale, stands characterized by a single main pulse of establishment in the 19^th^ or 20^th^ century along with remnant older trees and stands characterized by heterogeneous age structures with a high proportion of trees that established prior to the 19^th^ century recorded numerous tree deaths in the 1940s and marked peaks in released trees in the 1940s corresponding to the effects of the 1940s spruce beetle outbreak (Fig 3E–3F and 3H–3I). In contrast, none (0%) of the stands with pulses of establishment in the 19^th^ century that lacked remnant trees were affected by the 1940s outbreak as reflected by the lack of tree deaths in the 1940s and absence of a peak in growth releases in the 1940s (Fig 2B–2C). The pattern for stands characterized by a single main pulse of establishment in the 19^th^ or 20^th^ century with no remnant older trees is distinct from the patterns for other stand types in which 56% and 92% of stands, respectively, were affected by the 1940s spruce beetle outbreak (Chi-square = 19.4; *p* < 0.001). Stands affected by the outbreak also had a higher density of spruce at the time of sampling (Median = 1060/ha) than stands that were not affected (Median = 120/ha; Mann-Whitney Rank Sum Test *p* < 0.001).

**Fig 4:**
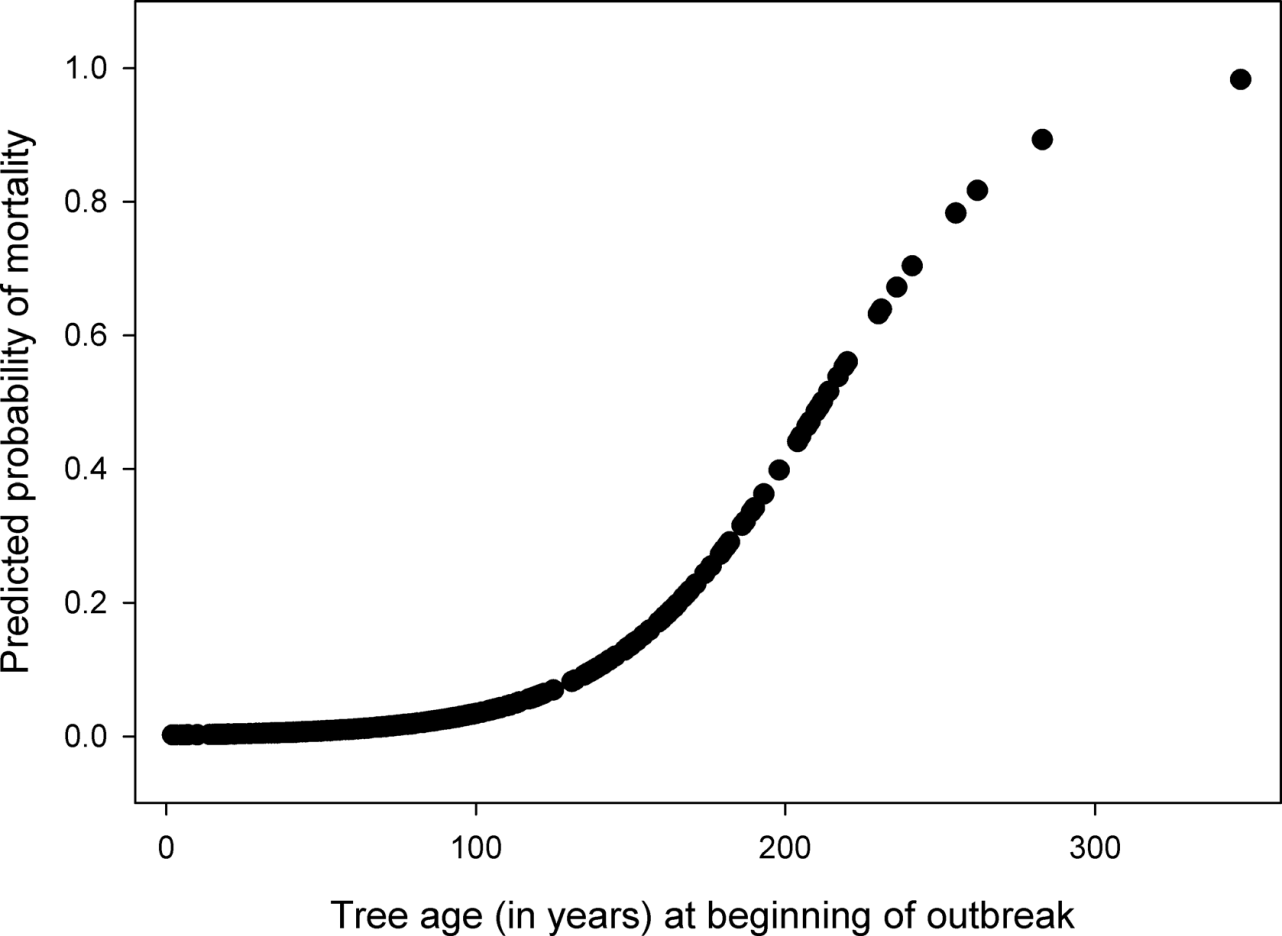
Logit model. Logit predicted probability of being killed during the outbreak as a function of tree age (years) at the beginning of the outbreak (1939). Tree age is based on pith, estimated pith, and minimum ages of 306 Engelmann spruce across all 32 sites.

At the scale of individual trees, age strongly determined mortality during the 1940s outbreak. Across all sites, spruce that died during the 1940s outbreak (between 1939 and 1949) established between 1804 and pre-1656, meaning that all were ≥ 128 years old during the outbreak (median age in 1939 of 199 years). In contrast, spruce that survived the 1940s outbreak had a broad range of ages and were overall much younger than the trees that died (median age in 1939 of 70 years). Probability of mortality during the outbreak increased with tree age (*p* < 0.001; Table 3; Fig 4):

LogitP=−6.321+(0.0298*Age in1939) (1)

**Table 3:** Details of logistic regression. Model based on mortality of 306 Engelmann spruce during the 1940s spruce beetle outbreak.

Independent Variable	Coefficient	Standard Error	Wald Statistic	*p*-value
Constant	-6.321	0.809	61.106	<0.001
Age in 1939	0.0298	0.00471	40.093	<0.001

Furthermore, stands that lacked a signal of the 1940s outbreak (including some stands > 100 years of at the time of the outbreak) had lower densities of spruce (0–1620, median 120 stems ≥ 10 cm dbh /ha) in comparison to stands that did show a signal of the 1940s outbreak (400–1820, median 1060 stems ≥ 10 cm dbh /ha; Mann-Whitney Rank Sum Test *p* <0.001).

## Discussion

The two main findings of this study are as follows. First of all, the severity of fires affects the degree to which susceptibility to subsequent outbreaks of spruce beetle is reduced. The broad-scale overlap between 19^th^ century fires and the 1940s spruce beetle outbreak was most likely due to spatial variation in severity of fires resulting in legacies of older trees either as scattered individuals or as remnant patches (Table 2), rather than either rapid tree growth and stand development on favorable sites, or beetle pressure overcoming constraints related to stand structure attributes. Second, under the relatively cool and wet climate of the 1940s (Table 1; Fig 2), stand-replacing fires of the preceding century resulted in stand structures that strictly constrained susceptibility to the 1940s outbreak.

The present findings are consistent with previous research that has found severe wildfires to reduce susceptibility to outbreaks of spruce beetle for c. 100 years [2,31–33]. However, in directly examining the effects of fire severity, we found that reduced susceptibility following fires is contingent on the severity of those fires. The research design of the current study specifically targeted stands that historical maps or remotely sensed data suggested had burned in the 19^th^ century and then had been affected by spruce beetle in the 1940s. The only stands that showed evidence of both disturbances were those in which fire in the 19^th^ century was of moderate or mixed severity and left numerous surviving trees, which were then killed in the outbreak. We could find no stands that had originated following high-severity, stand-replacing fire in the 19^th^ century (i.e. lacking remnant trees) and that were affected by the 1940s outbreak (Table 2). Indeed, tree age strongly predicted the likelihood of a tree being killed during the outbreak (Fig 4). The lack of evidence of outbreak in old stands with low spruce dominance may reflect limitations of dendroecological methods in detecting very low severity outbreaks (based on percentage of all trees killed), though it may also indicate stand structural constraints on susceptibility to outbreak. The finding that variations in fire severity controlled susceptibility to subsequent disturbances has broad significance in part because any remotely-sensed mapping of fires that does not adequately capture the presence or absence of remnant trees (e.g. Monitoring Trends in Burn Severity; MTBS) may not adequately represent the susceptibility of stand to post-fire disturbances.

Given the relative scarcity of fires during the early and mid-20^th^ century in the subalpine forests of western Colorado [32,48], data comparable to those presented in the current study are not available for the spruce beetle outbreak of the early 21^st^ century. Thus a direct comparison of how preceding fires have affected the recent outbreak is not feasible. However, the data that are available for a 2000s spruce beetle outbreak in nearby Grand Mesa National Forest indicate that relatively small, young trees have been attacked during the recent outbreak and that stand structural traits may not have been as constraining as expected [37]. In contrast, the data presented here indicate that the 1940s outbreak was very strongly controlled by stand structure as determined by preceding disturbance history. Observations of an apparent relaxation of the constraints of stand structural traits on spruce beetle outbreak in the 2000s outbreak in Grand Mesa National Forest [37] and the results of the current analysis are consistent with the prediction that under currently more favorable climate conditions for outbreaks (Table 1; Fig 2), the constraints imposed by tree and stand traits may be less effective. This can be explained by the fact that warm and dry conditions promote the growth of beetle populations and simultaneously stress host trees and compromise their defense mechanisms.

### Conclusions

Variations in disturbance severity and a changing climate present two important challenges to understanding disturbance interactions. The findings of the current study show that under the cooler and wetter conditions of the mid-20^th^ century, susceptibility to and spatial patterns of spruce beetle outbreak were most likely controlled by variations in severity of prior disturbance by fire. Even in ecosystems generally characterized by high-severity disturbances, variations in disturbance severity affect how disturbances interact. While methodologically more challenging, examining such variations can improve our understanding of disturbances interactions. A second important challenge is to better understand how disturbances interact in the context of climatic variability. The findings of the current study suggest that under the climatic conditions of the mid-20^th^ century, that were less favorable to spruce beetle outbreak than those of the early 21^st^ century, stand structure, as meditated by fire severity, strictly controlled susceptibility to and spatial patterns of the widespread spruce beetle outbreak. However, this relationship may be weakened during the outbreaks of the 21^st^ century and subsequent outbreaks, as climate change is likely changing how disturbances interact. For example, strong trends towards warmer minimum temperatures are likely to have important effects on beetle survival and reproduction, which will continue to affect how stand structure, as shaped by previous disturbances, affects susceptibility to outbreaks. Models of future forest development should consider the changing nature of disturbance interactions.
